# Transcutaneous vagus nerve stimulation during semantic verbal fluency tasks: a high-density diffuse optical tomography study

**DOI:** 10.3389/fnhum.2026.1964706

**Published:** 2026-10-05

**Authors:** Caitlin H. Illingworth, Sheharyar S. Baig, Jasmine Swalwell, Michal Holowacz, Samantha L. Dorney, Sabrina Di Lonardo Burr, Emilia Butters, Daniel Blackburn, Ali Ali, Arshad Majid, Li Su

**Affiliations:** 1 Department of Neuroscience, Sheffield Institute of Translational Neuroscience, University of Sheffield, Sheffield, United Kingdom; 2 School of Psychology, University of Sheffield, Sheffield, United Kingdom; 3 Department of Electrical Engineering, University of Cambridge, Cambridge, United Kingdom; 4 Department of Psychiatry, University of Cambridge, Cambridge, United Kingdom

**Keywords:** fNIRS, HD-DOT, neuromodulation, non-invasive stimulation, taVNS

## Abstract

**Introduction:**

Transcutaneous auricular vagus nerve stimulation (taVNS) is a promising adjunct for post-stroke language recovery. There are no established biomarkers for the neuromodulatory effects of taVNS. High-density diffuse optical tomography (HD-DOT) offers a non-invasive marker of task-related cortical activation. We explore HD-DOT in determining the effects of taVNS during a semantic verbal fluency task (VFT).

**Methods:**

Twenty-four healthy adult participants performed a semantic VFT in a block design, whilst HD-DOT was recorded across the bilateral prefrontal cortex. Participants received sham (earlobe) taVNS followed by active (tragus) taVNS. Statistical analysis was performed at a group level within condition (task–baseline) and between conditions (active taVNS vs. sham taVNS) using node-level and parcellation approaches.

**Results:**

Twenty-one participants (87.5%) met the signal quality threshold. At the uncorrected level, significant increases in task-related activity were seen in the triangular part of the left inferior frontal gyrus, during both the active and sham taVNS. Correcting for multiple comparisons, significant increases were seen in the left lateral orbitofrontal cortex during sham stimulation. No significant differences in oxygenated haemoglobin concentration increase were seen between active and sham taVNS.

**Discussion:**

Simultaneous recording of brain activity to taVNS during a semantic VFT was feasible and well tolerated. Task-related activation was seen in brain regions associated with language production and decision-making. Through this exploratory work, healthy adults do not demonstrate any significant enhancement of frontal cortical activation from taVNS. Future studies should combine whole-brain HD-DOT and taVNS in clinical cohorts.

## 1 Introduction

Aphasia is an acquired language disorder, characterised by difficulty in the ability to produce and/or comprehend language, associated with left hemispheric damage (Castro et al., 2023). Aphasia occurs in approximately 30% of stroke survivors (Berthier, 2005) and is one of the leading causes of post-stroke disability (Chen et al., 2025). The degree of aphasia recovery varies across individuals (Brady et al., 2016), with 30–43% of patients remaining affected long term (Bakheit et al., 2007). Aside from speech and language therapy, there are no adjunctive therapies available in the UK National Health Service (NHS) that can improve long-term recovery.

Vagus nerve stimulation (VNS) paired with rehabilitation enhances arm motor recovery after stroke (Dawson et al., 2021). Non-invasive or transcutaneous VNS to the auricular branch of the vagus nerve on the ear (taVNS) shows promising preliminary evidence in motor and balance recovery after stroke (Baig et al., 2023) with several ongoing trials investigating taVNS in stroke motor (Baig et al., 2025) and language recovery [TRANSLATE, NCT06403475]. Given VNS increases cerebral blood flow during verbal fluency tasks (VFTs) (Kunii et al., 2021) and taVNS can enhance second language vocabulary acquisition (Phillips et al., 2021; Miyatsu et al., 2024), taVNS has clinical potential in aphasia recovery.

A key barrier to clinical implementation of taVNS in language recovery is the ability to demonstrate cortical engagement during speech via scalable biomarkers (Burger et al., 2020). These biomarkers would enable clinicians to monitor neuromodulation, to support individually calibrated taVNS parameters for optimal aphasia intervention efficacy (Burger et al., 2020). Functional magnetic resonance imaging (fMRI) studies have shown that taVNS activates brainstem projections of the vagus nerve (e.g., locus coeruleus and the nuclei tractus solitarius) and can modulate cortical frontoparietal networks (Burger et al., 2020; Badran, B. et al., 2018). However, the cost and contraindications of fMRI make it impractical to use to track longitudinal responses to taVNS, and the incompatibility of fMRI with many electronic devices make concurrent fMRI and non-invasive brain stimulation challenging (Hartwig et al., 2017). On the other hand, while EEG is able to capture viable markers of taVNS induced neuromodulation (Camargo et al., 2025; Gianlorenco et al., 2022), this imaging is highly susceptible to muscle and motion artifacts, making recording during speech challenging.

Functional near-infrared spectroscopy (fNIRS) has emerged as a portable tool to assess neural activity thar can overcome some of fMRI’s barriers. FNIRS uses near-infrared light to determine cortical concentrations of oxygenated (HbO) and deoxygenated haemoglobin (HbR). Several studies have used low-density fNIRS alongside language tasks to assess cortical activation in healthy and clinical populations (Li et al., 2022; Tupak et al., 2012; Herrmann et al., 2017). Whilst traditional systems were low density (16–64 channels), high-density fNIRS (HD-fNIRS) devices incorporate densely packed source-detector optodes that, when reconstructed with anatomical priors yield 3D reconstructions through diffuse optical tomography (DOT), with a spatial resolution approaching that of fMRI (Eggebrecht et al., 2012).

Our work has previously focused on investigating the effects of taVNS during motor task and cognitive tasks using HD-DOT (Baig et al., 2026; Holowacz et al., 2026). No previous studies have evaluated HD-DOT signals with verbal fluency tasks. These tasks have been shown to engage the left frontal and temporal regions, namely Broca’s area, being highly left lateralised among healthy monolinguals and are commonly used across neurological cognitive tests (Heim et al., 2008; Biesbroek et al., 2021; Biesbroek et al., 2016). The present study aims to assess the cortical and behavioural effect of taVNS in healthy volunteers during a semantic verbal fluency task given its clinical utility and well-defined region of response. Given the previous neuromodulatory effect seen in taVNS, we hypothesise that there will be a greater increase in oxygenated haemoglobin concentration during the verbal fluency tasks when participants receive active stimulation, especially among the left frontal regions including Broca’s area (Biesbroek et al., 2021; Biesbroek et al., 2016).

This will provide insight into the biomarkers of taVNS neuromodulation in a healthy population, acting as a baseline of expected effects to be used later in clinical populations.

## 2 Materials and methods

### 2.1 Participants

A total of 24 healthy adults (13 female, *M*_age_ = 24.8, SD = 5.62, 4 bilinguals) were recruited through convenience sampling from staff and students at the University of Sheffield. Participants were excluded if they had an open wound on scalp/head or contraindications to taVNS (see (Baig et al., 2026; Holowacz et al., 2026). Ethical approval for the study was granted by the University of Sheffield Ethics Board (Ethics Reference: 058405) and have therefore been performed in accordance with the ethical standards laid down in an appropriate version of the 1965 Declaration of Helsinki.

### 2.2 Experimental design

This employed a within-subjects block design to investigate the effects of taVNS on cortical activity. Demographic information was collected, and participants were checked for taVNS contraindications. Each participant completed a set of semantic verbal fluency tasks and an N-Back task under sham then active taVNS (Holowacz et al., 2026). The verbal fluency task results are the focus of the current study.

### 2.3 Verbal fluency tasks

Participants completed three verbal fluency trials during sham stimulation, followed by three verbal fluency trials with active stimulation. Trials consisted of a pseudo-jitter rest period of 30 s (±2 s) followed by a 60-s verbal fluency task (Figure 1). During rest periods, participants recited the days of the week to help isolate neural markers of cognitive engagement rather than motor speech-activation. During verbal fluency tasks, participants were instructed to verbally generate as many English words as possible from the category written on screen in 1 min, reflecting the VFT duration during clinical cognitive assessments (Cintoli et al., 2024). To minimise learning or repetition effects, no prompts were repeated within participants. Two distinct sets of categories were used across the two stimulation conditions (Set A: Animals, Vegetables, Sports; Set B: Clothing, Fruit, Professions), with the order counterbalanced across participants. Verbal responses were written down by researchers during the task for subsequent analysis.

**Figure 1:**
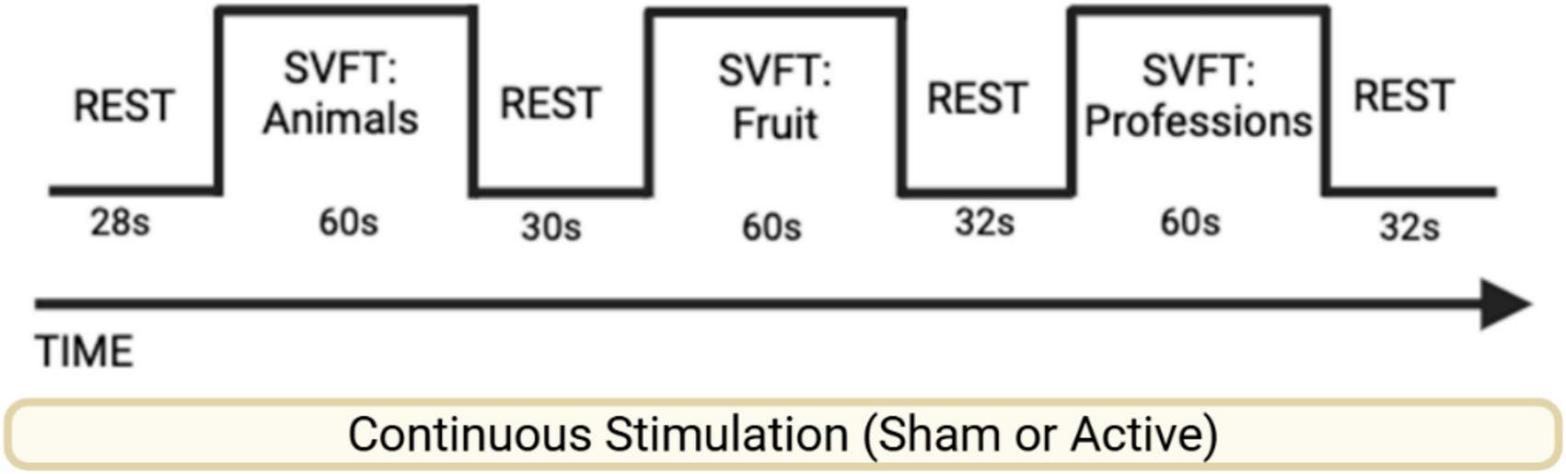
Block design for one experimental condition (3 trials). SVFT, semantic verbal fluency task.

### 2.4 Experimental set-up

During trials, the semantic category was presented on screen, and a fixation cross was displayed during rest tasks. All instructions and stimuli were presented using PsychoPy (v2024.2.24) on a 14-inch laptop positioned approximately 60 cm from the participant for the duration of the task. Data collection took place in a quiet room at the University of Sheffield or in the Royal Hallamshire Hospital Biomedical Research Centre.

### 2.5 HD-DOT acquisition

The HD-DOT data was collected using a LUMO (Gowerlabs Ltd., UK) high-density continuous wave fNIRS system with 12 tiles placed in a bilateral prefrontal cortex array (Figure 2a). The LUMO system consists of multidistance overlapping channels, enabling the dissociation of hemodynamic data from the scalp (10–12 mm channels) and the cortex (12–42.5 mm) (Butters et al., 2025). Each tile contained three dual-wavelength LED sources (735|850 nm) and four photodiode detectors, for a total of 36 sources and 48 detectors, where each source forms a channel with each detector for a total possible 1728 channels (~464 between 10 and 42.5 mm).

**Figure 2:**
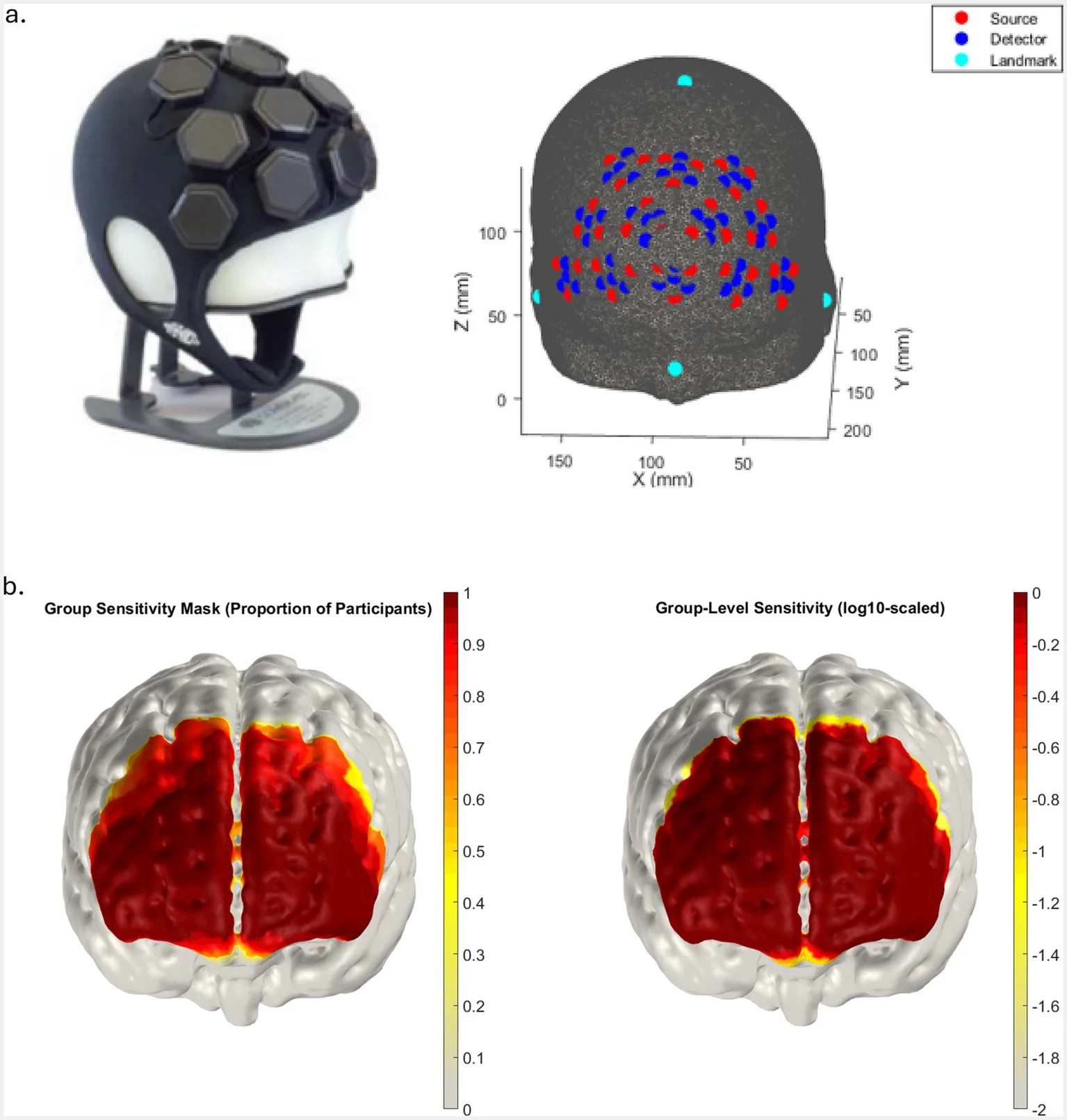
**(a)** Source-detector registration to template brain showing locations of optodes. **(b)** Sensitivity mask for HD-DOT illustrating average sensitivity across all participants. Node sensitivity is defined as using Uchitel et al. (2022) thresholds for detecting changes in HbO and HbR. Picture of 12 tile LUMO cap (source: https://www.gowerlabs.co.uk/lumo).

### 2.6 taVNS delivery

A NurosymTM device (Nurosym Ltd., United Kingdom) delivered taVNS stimulation at the device’s pre-set parameters which have been used in prior clinical studies; 25 Hz, pulse width of 250 μs. These stimulation parameters were consistent between conditions. Active taVNS was delivered at the left tragus whilst sham stimulation was delivered at the left earlobe which lacks vagus nerve innervation (See (Baig et al., 2026; Holowacz et al., 2026). Stimulation was delivered via the left ear to reduce the risk of cardiac events (Forte et al., 2022). The tragus was chosen as the active stimulation site in line with ongoing clinical trials assessing tragus-taVNS in stroke recovery [TRANSLATE, NCT06403475; 9], and for ease of stimulator attachment. Intensity was matched at the mid-point between the perceptual threshold and the pain threshold.

Stimulation began immediately before each block of trials, continuing through both task and rest. Each stimulation condition therefore lasted approximately 15 min (5 min during task instructions and practise, 5 min during VFT trials, and 5 min during N-back trials with the order of tasks counterbalanced). There was no washout period between stimulation conditions, meaning that active stimulation followed immediately after sham was completed. Blinding effectiveness to the stimulation condition was not assessed.

### 2.7 Behavioural analysis

To investigate the potential effect of taVNS on cognitive performance, the mean number of responses across the three VFTs within each condition was calculated for each participant. Paired *t*-tests were conducted to analyse the change in the mean number of responses between stimulation conditions.

To investigate the effect of stimulation on the relationship between activation and performance, an exploratory correlational analysis was conducted between participants’ Δ HbO concentration for each AAL2 parcel and mean VFT score for each stimulation condition using a Pearson correlation. FDR correction was applied to control for Type I error (Benjamini and Hochberg, 1995). Both corrected and uncorrected *p*-values are reported.

### 2.8 HD-DOT preprocessing

We implemented the same analysis pipeline as used in our previous work (Holowacz et al., 2026). Data preprocessing was conducted using MATLAB R2023b (MathWorks, Natick, MA), using a custom pipeline that incorporated functions from Homer2 (Huppert et al., 2009) and the DOT-HUB toolbox (https://github.com/DOT-HUB/DOT-HUB).

Raw light intensity signals were first converted to changes in optical density (ΔOD) using the Homer2 toolbox (Huppert et al., 2009). Channels were excluded if the raw light intensity was outside [0 1e11], the signal-to-noise ratio fell below 12, or source-detector distances were over 100 mm (Butters et al., 2025). Motion artefacts were automatically detected at the channel level using amplitude and standard deviation thresholds (0.1 and 50, respectively) and corrected via HmrMotionCorrSplineSG spline interpolation and Savitzky–Golay filtering (10-s window, spline parameter = 0.99) (Jahani et al., 2018). Optical density data was converted to concentration changes in HbO and HbR using the hmrOD2Conc function with a differential pathlength factor of 6. Local short-channel regression (0–12 mm) followed by a third-order Butterworth bandpass filter (0.01–0.1 Hz) was implemented to reduce contamination from superficial signal. Task-evoked hemodynamic responses were extracted using block averaging, time-locked to stimulus onset. Averaging windows spanned −5 to +60 s, with −5 to 0 s defined as the baseline and 10 to 20 s as the task period. The task period of 10–20 s was chosen as it captured peak activation while avoiding confounding deactivation when participants disengaged after exhausting their responses.

### 2.9 Image reconstruction

For each participant, a map of cortical oxygenation (HbO and HbR) was reconstructed using a tetrahedral grey matter mesh based on the standard MNI-152 template head model (Maintz and Viergever, 1998). For reconstruction, concentration changes in both chromophores were converted to optical densities using DOTHUB_hmrConc2OD. To reconstruct the optical data, a forward model of light propagation was calculated using the DOTHUB_make_Jacobian function. From this, a zeroth-order Tikhonov regularised inversion was performed using a regularisation hyperparameter of 0.01.

### 2.10 Node-level statistical analysis

Participants were excluded from further analysis if <1/3 of channels within 10–42.5 mm met pruning criteria (Fiske et al., 2022). The average HbO concentration for each reconstructed node across all participants at baseline (−5–0 s) and task (10–20 s) was extracted for each condition. For within-condition analysis paired *t*-tests were to compare node-level changes in HbO during the task versus baseline periods. For between-condition analysis, the mean change in HbO concentration from baseline to task was calculated for each condition and compared between conditions. The derived *t*-stats were then plotted on the cortical reconstruction, with only nodes with *p* < 0.05 plotted. To control for multiple comparisons, an FDR correction was performed (Benjamini and Hochberg, 1995).

### 2.11 Parcellation analysis

Reconstructed data for each participant was parcellated using the AAL2 atlas (Rolls et al., 2015), assigning each grey matter mesh node to one of 15 corresponding frontal regions of interest (see Appendix A). Based on work by Uchitel et al. 2022, a node in the grey matter mesh was defined as sensitive if its sensitivity in the Jacobian matrix exceeded 5% of the maximum value of the normalised Jacobian for both wavelengths (Figure 1B, Uchitel et al. 2022). For each participant, parcels were only included in subsequent averaging and analysis if over 50% of the grey matter mesh nodes within were sensitive to both HbO and HbR changes. A single time series was then calculated for each parcel by averaging the time series of all sensitive nodes within that parcel.

For each parcel, task-evoked changes in HbO and HbR were calculated based on baseline and task windows. Only ΔHbO was analysed in the present study due to its higher signal-to-noise ratio (Tachtsidis and Scholkmann, 2016). Mean HbO concentrations at baseline and task activation window were extracted for each participant, parcel, and condition, providing the inputs for group-level statistical analyses. Paired *t*-tests were conducted for all parcels between the group average baseline and active mean HbO. Multiple comparisons were corrected using the false discovery rate (FDR) method (Benjamini and Hochberg, 1995). Both corrected and uncorrected *p*-values are reported.

## 3 Results

### 3.1 Participant characteristics

Participant recruitment took place between April–July 2025. Three participants were excluded across conditions for the node-level analysis due to <⅓ channels meeting the pruning criteria. The characteristics for each cohort are reported in Table 1.

**Table 1:** Participant characteristics.

Characteristic	Total cohort	Cohort after quality exclusion
	*n* (%) or *M* (SD)	*n* (%) or *M* (SD)
N	24	21
Age (years)	24.8 (5.62)	24 (3.2)
Sex		
Female	13 (54.2%	12 (57.1%)
Male	11 (45.8%)	9 (42.9%)
Race/ethnicity		
White	20 (83.3%)	17 (81.0%)
South Asian	1 (4.2%)	1 (4.8%)
Chinese	1 (4.2%)	1 (4.8%)
Mixed race: White and Asian	1 (4.2%)	1 (4.8%)
Mixed race: White and Black	1 (4.2%)	1 (4.8%)
First language		
English	20 (83.3%)	18 (85.7%)
Mandarin	1 (4.2%)	1 (4.8%)
French	1 (4.2%)	1 (4.8%)
Spanish	1 (4.2%)	0 (0%)
Polish	1 (4.2%)	1 (4.8%)
Hair colour		
Brown	18 (75.0%)	15 (71.4%)
Blonde	2 (8.3%)	2 (9.5%)
Black	2 (8.3%)	2 (9.5%)
Red	1 (4.2%)	1 (4.8%)
No hair	1 (4.2%)	1 (4.8%)
Handedness		
Right	21 (87.5%)	18 (85.7%)
Left	3 (12.5%)	3 (14.3%)

### 3.2 TaVNS side effects

Stimulation intensity ranged from 11.2–24.8 (mA) during sham stimulation (*M* = 18.7 mA, SD = 4.2), and 13.6–25.6 (mA) during active stimulation (*M* = 19.5 mA, SD = 4.3). There was no significant difference in the stimulation intensity between conditions (*t*(23) = −1.15, *p* = 0.26). At the end of each session, participants reported any stimulation side effects they experienced. Four participants provided no response. Of the 20 survey responses recorded, 10 noted some discomfort during taVNS stimulation, though these were all mild and no participants withdrew from the stimulation. None of the side effects persisted beyond the stimulation. An overview of reported side effects is shown in (Table 2).

**Table 2:** Side effects reported by participants from stimulation.

Number of participants reporting side effect	Stimulation side effect
4	Local pinching sensation (worse during active stimulation for 2 participants)
2	Local itching sensation (only during active stimulation for 1 participant)
2	Local mild pain/discomfort
2	Local stinging/burning sensation (only during active stimulation for 1 participant)
2	Local vibration

### 3.3 Task performance

Two participants were excluded from the performance analysis due to missing verbal responses (*N* = 22). Participants’ VFT performance across the stimulation conditions is shown in Figure 3. A paired-samples *t*-test found no statistically significant difference in the mean number of words produced during the VFT trials between the sham (*M* = 20.74, SD = 1.45) and active conditions (*M* = 21.78, SD = 1.74) (*t*(21) = 1.23, *p* = 0.23). As such, we found no evidence of changes in the VFT performance when receiving taVNS stimulation.

**Figure 3:**
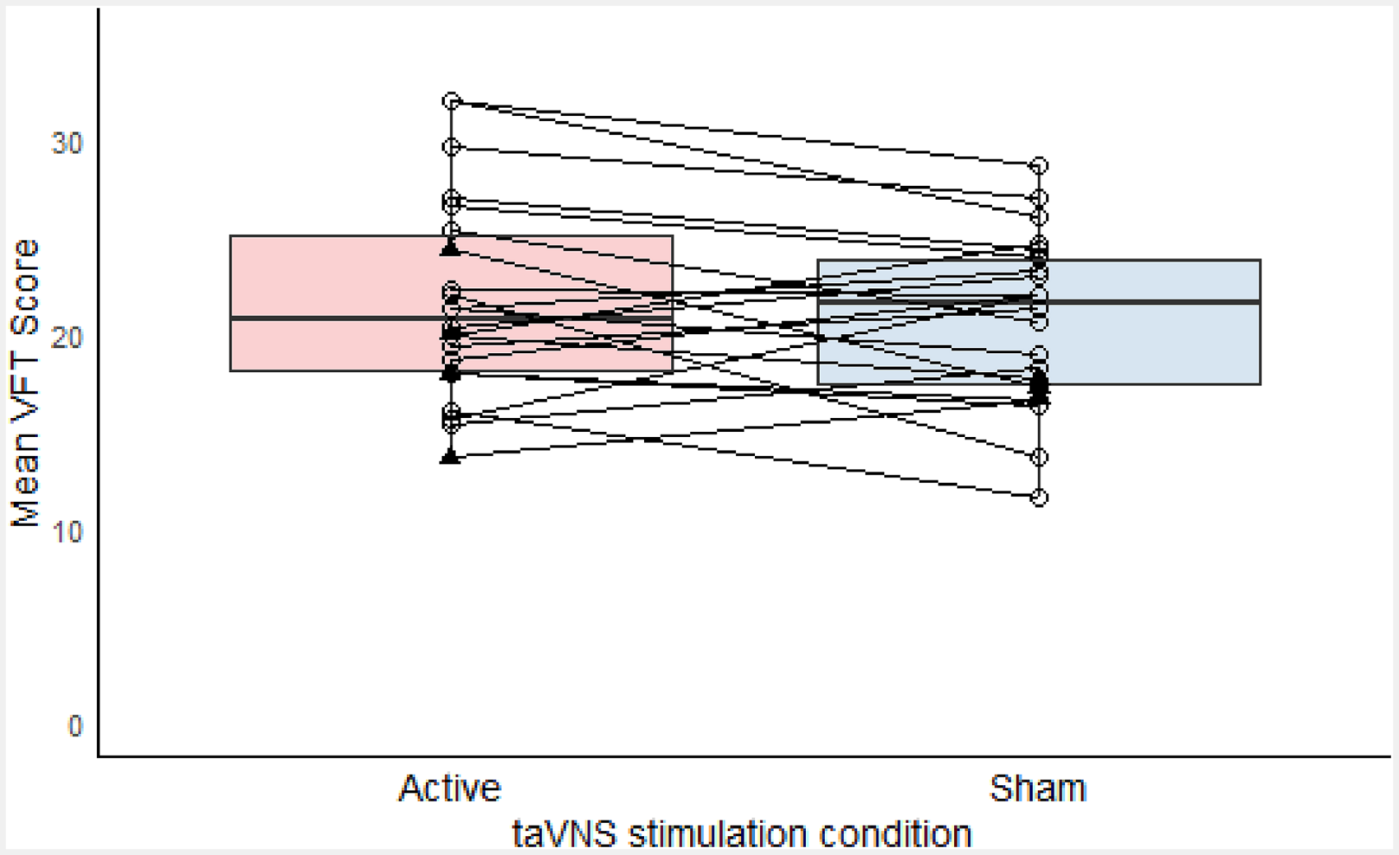
Mean number of words produced in each stimulation condition. Box denotes first and third quartiles in the interquartile range (IQR) with the median depicted as the horizontal line within. Whiskers denote range of values within 1.5 * IQR. Points denote individual participant performance, with lines connecting between performance for each condition. Shape denotes whether English was the participant’s first language, hollow circle = English first language, black triangle = English second language.

Exploratory Pearson’s correlational analysis identified positive associations between regional activation (ΔHbO) and VFT performance in the left anterior orbital gyrus (*r* = 0.53, 95% CI [0.13, 0.78], *n* = 21, *p* = 0.014) and the left dorsolateral superior frontal gyrus (*r* = 0.48, 95% CI [0.08, 0.75], *n* = 22, *p* = 0.021) only during active stimulation. However, these associations did not survive FDR correction for multiple comparisons (see Appendix Figure B1).

### 3.4 Group-average maps

Group-level HbO and HbR activation maps were created to visualise the spatial pattern and degree of task-related activity under both sham and active taVNS conditions during the task period (Figure 4). Both tasks showed a similar distribution of increased HbO with widespread bilateral frontal activity, with HbO concentration appearing higher in the sham condition. HbR maps show the expected reciprocal concentration changes.

**Figure 4:**
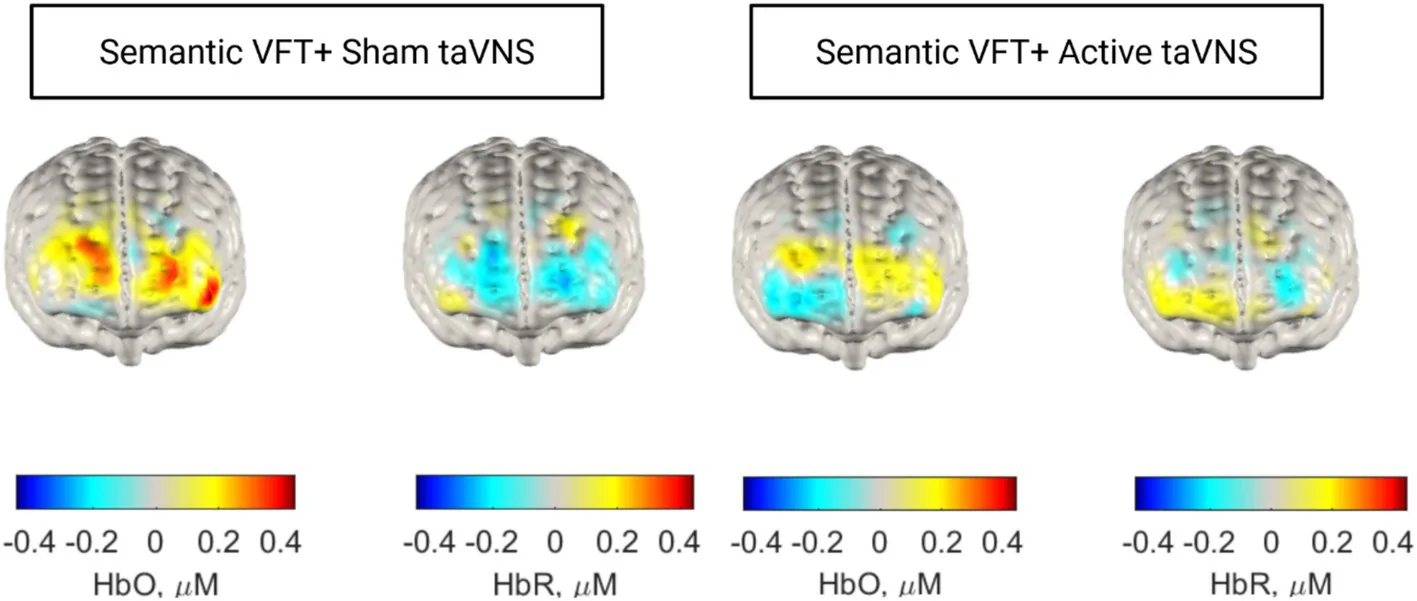
Group level average HbO and HbR concentration during VFTs 10–20 s post-stimulus onset projected on to an MNI cortical surface.

### 3.5 Statistical analysis of taVNS on HD-DOT

To investigate the effect of taVNS on cortical activation, paired *t*-tests were conducted within each condition to compare node-level changes in the task versus baseline periods (Figure 5). When comparing the within-condition maps seen in the active and sham taVNS, participants exhibit more diffuse bilateral activation when receiving sham stimulation, while activation appears more focalised to the left inferior frontal gyrus in the active stimulation condition. This is also reflected in the between-condition map, wherein a cluster of relatively reduced activation was seen bilaterally, especially in the left posterior frontal region. However, no node-level differences survived FDR correction.

**Figure 5:**
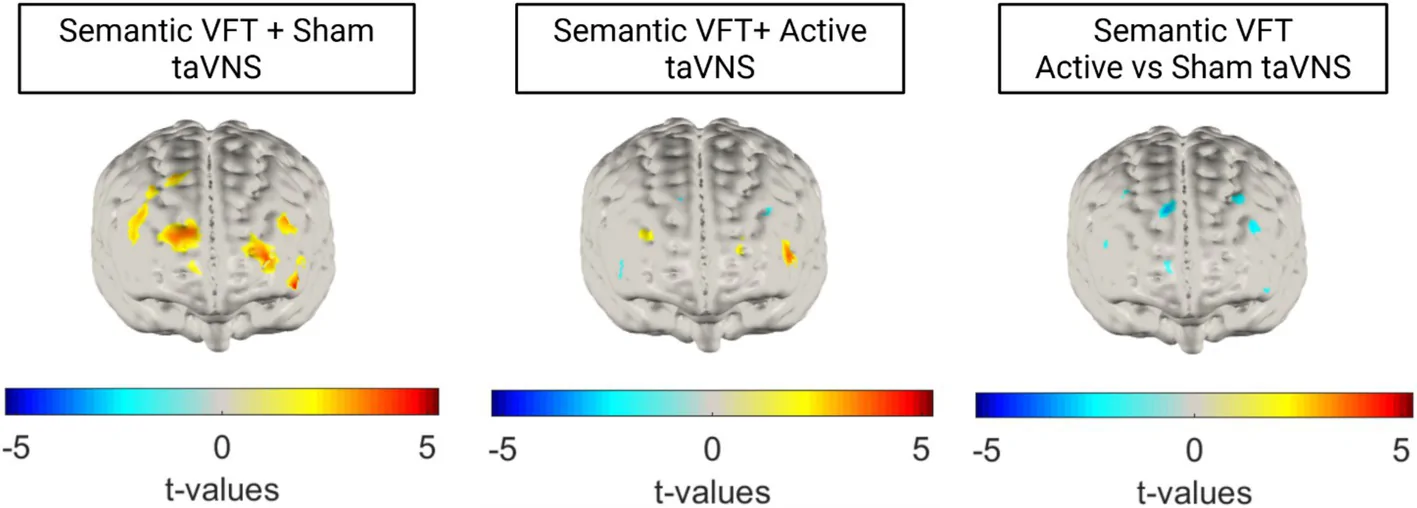
Cortical surface reconstruction of t-statistic maps comparing the block-averaged HbO concentration at baseline (−5 to 0 s pre-task) vs. task-peak (10–20 s) at the node-level. Only nodes with uncorrected *p* < 0.05 are shown. No nodes survived FDR correction.

### 3.6 Parcellation analysis

Reconstructions were parcellated into anatomical brain regions in the sensitive areas of the AAL2 atlas and the average HbO time series in each parcel was calculated. Parcels with significant task related increases or decreases in HbO at the uncorrected level are shown in Table 3 (see Appendix for full results).

**Table 3:** Parcel-wise statistical comparison of HbO concentration changes during verbal fluency tasks and between tasks.

	Parcels	*N*	Baseline HbO (μM)		Task HbO (μM)		Δ HbO (μM)	*p*	FDR *p*	Cohen’s *d*
			*M*	SD	*M*	SD				
Sham	Left Superior frontal gyrus_2	23	−4.70E-18	1.31E-17	0.07	0.13	0.07	**0.02**	0.08	0.52
	Left Middle frontal gyrus_2	22	9.73E-20	7.72E-18	0.05	0.09	0.05	**0.02**	0.08	0.53
	Left Inferior frontal gyrus, triangular part	17	1.05E-18	1.07E-17	0.10	0.13	0.10	**0.01**	0.05	0.75
	Left Lateral orbital gyrus	24	4.74E-19	8.45E-18	0.10	0.14	0.10	**0.002**	0.03*	0.71
Active	Left Inferior frontal gyrus, triangular part	17	3.08E-19	4.48E-18	0.06	0.09	0.06	**0.02**	0.23	0.66

	Parcels	*N*	Change in Active		Change in Sham		Δ *Diff* (μM)	*p*	FDR *p*	Cohen’s *d*
			*M*	SD	*M*	SD				
Active vs. Sham	Left lateral orbital gyrus	24	0.01	0.15	0.10	0.14	−0.09	**0.04**	0.57	−0.44

N.B. Only parcels with an uncorrected *p* < 0.05 are reported. *Denotes *p* <.05 after FDR correction. Bold denotes *p* <.05 without correcting for multiple comparisons.

During sham taVNS, there were significant task-related increases at the uncorrected level in the left superior frontal gyrus, middle frontal gyrus, inferior frontal gyrus (triangular part) and lateral orbital gyrus compared to baseline. After FDR correction, significant increases in activation in the left lateral orbital gyrus persisted.

During active taVNS, there was a significant task-related increase in the left inferior frontal gyrus (triangular part) at the uncorrected level, which did not survive FDR correction. When comparing stimulation conditions, the left lateral orbital gyrus left exhibited a significantly greater HbO concentration increase during the sham stimulation compared to active stimulation. However, no regions were significantly different between the active versus sham conditions after FDR correction.

## 4 Discussion

This exploratory pilot study found that simultaneous delivery of taVNS whilst recording haemodynamic response using HD-DOT was feasible and well tolerated, with no participants ending the session early. HD-DOT was able to detect task-related activation, which at the uncorrected level, localised to the left Broca’s area. There was no significant difference in task performance, nor HbO concentration change during VFTs with active taVNS compared to sham. During active stimulation, we found significant positive correlations between performance and activation in the left anterior orbital gyrus and the left dorsolateral superior frontal gyrus, associated with working memory (du Boisgueheneuc et al., 2006) and language (Dick et al., 2019). Additionally, uncorrected t-stat maps indicate a more focalised activation during active taVNS. In sum, while these exploratory findings point toward a potential modulation of task-related activity, the effect was not great enough nor robust enough to survive correcting for multiple comparisons. This may be due to the small sample of healthy participants used, or a true lack of effect on performance and activation.

### 4.1 Functional neuroimaging of language function

Our findings align with a previous review of fMRI studies in healthy individuals showing consistent activation in the left inferior frontal gyrus (Price, 2010). Given that our system recorded exclusively from a frontal cortex array and near-infrared light does not penetrate much beyond 2 cm into the brain (Pinti et al., 2020), it was not expected that the subcortical or temporal regions involved with language processing and decision-making seen in fMRI research would be captured (Wagner et al., 2014). As such any changes within these regions were missed, limiting the full evaluation of taVNS linguistic modulation. Whilst the HbO concentration in the left superior, middle and inferior frontal gyrus significantly increased in the task-baseline comparison in the sham task, these did not survive corrections for multiple comparisons, possibly due to the sample size, the natural variability in subject-specific activation patterns, and the methodology used in HD-DOT analysis compared to fMRI.

FNIRS has been utilised to assess cortical responses during language tasks in healthy individuals and those with aphasia. Healthy individuals commonly exhibit increased HbO concentrations in the left prefrontal cortex during verbal fluency tasks (Tupak et al., 2012). When using fNIRS, there is some variability regarding how left lateralised this activity is (Onishi et al., 2019), reflected in our study. Patients with aphasia have been shown to exhibit greater activation across the left IFG, middle temporal gyrus and superior temporal gyrus during a naming task, with the latency of the HbO concentration in the left supramarginal gyrus correlating with performance (Li et al., 2022). Further, Gilmore et al. (2021) used fNIRS during semantic feature analysis and picture naming tasks in individuals with aphasia and healthy controls, finding increased activation outside the language network regions seen in the healthy controls. When investigating activation using fNIRS, individuals with aphasia are likely to involve more domain general regions to complete language tasks, with HbO change speed as well as the intensity of HbO acting as useful markers to investigate for stimulation efficacy.

### 4.2 Functional imaging of taVNS

FMRI studies show inconsistent taVNS-related activation, which may relate to different stimulators and stimulation settings (Matsuoka et al., 2025). Common areas of increased activation are the left prefrontal cortex (Badran, B. et al., 2018; Badran, BW. et al., 2018), insula (left, bilateral) and decreased activation in the hippocampus (Badran, BW. et al., 2018). Other regions include the locus coeruleus and nucleus tractus solitarius (brainstem projections of the vagus nerve (Butt et al., 2020). These studies have investigated the direct effects of stimulation rather than taVNS task-related modulation. This is significant as the current paradigm for VNS-related neuroplasticity is the pairing of stimulation with a specific action to promote task-specific plasticity (Meyers et al., 2018).

FNIRS affords a more naturalistic setting for assessments of language function during overt speech production than fMRI (Scarapicchia et al., 2017) and allows for the concurrent use of all clinical electronic devices such as taVNS. Despite this, there are very few studies of fNIRS and taVNS (Zhu et al., 2024; Gao et al., 2025; Li et al., 2025; Wang et al., 2024; Wang et al., 2025; Wang et al., 2023) and none have previously used VFT performance as an outcome measure. One previous study using low-density (11-channel) fNIRS in individuals with an implanted vagus nerve stimulator for epilepsy found HbO concentration increases in the frontal cortices were greater when VNS was paired with a verbal fluency task, but not when VNS was delivered alone (Kunii et al., 2021). Illustrating the importance of assessing neuromodulation during task-paradigms, with implications for language rehabilitation. Whilst the present study did not show stimulation-related increases in haemodynamic response, the potential to use a device such as HD DOT to accurately monitor neuromodulation is critical to optimising intervention efficacy. Our inability to replicate this finding may be explained by differences in the study cohort (high-density vs. low-density fNIRS, healthy volunteers vs. people with epilepsy, non-invasive vs. invasive VNS). It is possible that haemodynamic responses to verbal fluency tasks are already optimised in healthy volunteers and cannot be acutely enhanced by taVNS, though further research is needed to determine this.

### 4.3 Limitations

There are some limitations to the present study. First, we did not use a subject-specific registration of HD-DOT to head anatomy/shape which may dilute some group-level comparisons in a small sample size (Srinivasan et al., 2024), which may explain the discrepancy we see in our results relative to fMRI. Nonetheless, the lack of subject-specific anatomy was offset by standardised cap placement and the within-subject comparison in a single session. Second, given the possibility of sustained or delayed effects of taVNS (Atanackov et al., 2025), we could not counterbalance the order of conditions: the active taVNS task always followed the sham taVNS task. Whilst this prevents a carryover effect of taVNS and ensures identical cap placement across tasks for each participant, there is a potential order effect, including a practise effect/fatigue, attenuating task-related activation. This may explain why the left lateral orbitofrontal gyrus, an area involved in goal pursuit and motivation (Rolls, 2023), increased in the sham (first) task but not the active taVNS (second) task. Additionally, the reduced activation seen in the active stimulation condition may reflect tiredness or disengagement after already completing the sham trials. Future work should investigate the effect of the number of VFT trials on task activation and performance, to help disentangle the effect of stimulation from fatigue.

Third, the effects of varying stimulation amplitude, frequency, duration, anatomical site (cymba concha vs. tragus) (Yakunina et al., 2017) and laterality of stimulation were not studied. Given that cymba concha has increased vagus nerve innervation relative to the tragus (Peuker and Filler, 2002), taVNS may have a greater neuromodulatory effect that could be detected using HD-DOT if this site was used, though the difference in effect of these stimulation sites is contested (Borges et al., 2021). There is evidence that there is an inverted U-shaped relationship between both current amplitude and current frequency and the response to invasive VNS (Baig et al., 2022), and bilateral taVNS remains under researched regarding its impact on cortical activation relative to unilateral stimulation (Sun et al., 2025). Future studies using HD-DOT systematically modulating stimulation parameters could determine whether specific factors modulate haemodynamic response and whether anatomical site effects this. Fourth, the analysis considered the mean difference in activation during a task window vs. baseline; with advances in the analysis of HD-DOT data, future analytic approaches may include a generalised linear model as in studies of functional MRI (von Lühmann et al., 2020). Finally, the cohort had a mixture of left- and right-handed individuals and included some individuals for whom English was not their first language; therefore, the semantic verbal fluency-related activation may not have localised uniformly across participants (See Appendix D) (Wroblewski et al., 2017).

## 5 Conclusion

This exploratory study investigated the effect of taVNS on task-related HD-DOT recorded brain activity and performance during verbal fluency tasks, to assess the potential of this approach for future studies in clinical populations, and evidence of neuromodulation. While HD-DOT was able to detect task-related activation across the frontal language regions, we found no evidence of neuromodulation after correcting for multiple comparisons. We speculate that this non-significant difference reflects the sample population (i.e., healthy cohort), small sample size, and study design restrictions. However, a genuine absence of an acute taVNS effect remains possible and warrants further research. This work is foundational for future studies of HD-DOT in the assessment of individuals with acquired language disorders, such as post-stroke aphasia, and for the investigation of non-invasive brain stimulation.

## Data Availability

The raw data supporting the conclusions of this article will be made available by the authors, without undue reservation.

## References

[ref1] Atanackov P. Peterlin J. Derlink M. Kovačič U. Kejžar N. Bajrović F. F. (2025). The acute effects of varying frequency and pulse width of transcutaneous auricular vagus nerve stimulation on heart rate variability in healthy adults: a randomized crossover controlled trial. Biomedicine 13: 700. doi: 10.3390/biomedicines13030700, 40149675 PMC11940630

[ref2] Badran B. Dowdle L. Mithoefer O. Badran B. W. Dowdle L. T. Mithoefer O. J. . (2018). Neurophysiologic effects of transcutaneous auricular vagus nerve stimulation (taVNS) via electrical stimulation of the tragus: a concurrent taVNS/fMRI study and review. Brain Stimul. 11, 492–500. doi: 10.1016/j.brs.2017.12.009, 29361441 PMC6487660

[ref3] Badran B. W. Mithoefer O. J. Summer C. E. LaBate N. T. Glusman C. E. Badran A. W. . (2018). Short trains of transcutaneous auricular vagus nerve stimulation (taVNS) have parameter-specific effects on heart rate. Brain Stimul. 11, 699–708. doi: 10.1016/j.brs.2018.04.004, 29716843 PMC6536129

[ref4] Baig S. S. Illingworth C. H. McQueen B. Gibbons A. Ravenscroft J. Morton C. . (2026). Transcutaneous Vagus nerve stimulation during motor activity in healthy volunteers: a high-density diffuse optical tomography study. Brain Sci. 16:146. doi: 10.3390/brainsci16020146, 41750147 PMC12938713

[ref5] Baig S. S. Kamarova M. Ali A. Su L. Dawson J. Redgrave J. N. . (2022). Transcutaneous vagus nerve stimulation (tVNS) in stroke: the evidence, challenges and future directions. Auton. Neurosci. 237:102909. doi: 10.1016/j.autneu.2021.102909, 34861612

[ref6] Baig S. S. Kamarova M. Bell S. M. Ali A. N. Su L. Dimairo M. . (2023). tVNS in stroke: a narrative review on the current state and the future. Stroke 54, 2676–2687. doi: 10.1161/STROKEAHA.123.043414, 37646161

[ref7] Baig S. S. Mooney C. McKendrick K. Duffy K. E. M. Ali A. N. Redgrave J. N. . (2025). TRanscutaneous lImb reCovEry post-stroke (TRICEPS): study protocol for a randomised, controlled, multiarm, multistage adaptive design trial. BMJ Open 15:e092520. doi: 10.1136/bmjopen-2024-092520, 40139703 PMC11950934

[ref8] Bakheit A. M. Shaw S. Barrett L. Wood J. Carrington S. Griffiths S. . (2007). A prospective, randomized, parallel group, controlled study of the effect of intensity of speech and language therapy on early recovery from poststroke aphasia. Clin. Rehabil. 21, 885–894. doi: 10.1177/0269215507078486, 17981847

[ref9] Benjamini Y. Hochberg Y. (1995). Controlling the false discovery rate: a practical and powerful approach to multiple testing. J. R. Stat. Soc. B 57, 289–300.

[ref10] Berthier M. L. (2005). Poststroke aphasia: epidemiology, pathophysiology and treatment. Drugs Aging 22, 163–182. doi: 10.2165/00002512-200522020-00006, 15733022

[ref11] Biesbroek J. M. Lim J.-S. Weaver N. A. Arikan G. Kang Y. Kim B. J. . (2021). Anatomy of phonemic and semantic fluency: a lesion and disconnectome study in 1231 stroke patients. Cortex 143, 148–163. doi: 10.1016/j.cortex.2021.06.019, 34450565

[ref12] Biesbroek J. M. van Zandvoort M. J. Kappelle L. J. Velthuis B. K. Biessels G. J. Postma A. (2016). Shared and distinct anatomical correlates of semantic and phonemic fluency revealed by lesion-symptom mapping in patients with ischemic stroke. Brain Struct. Funct. 221, 2123–2134. doi: 10.1007/s00429-015-1033-8, 25939335 PMC4853441

[ref13] Borges U. Pfannenstiel M. Tsukahara J. Laborde S. Klatt S. Raab M. (2021). Transcutaneous vagus nerve stimulation via tragus or cymba conchae: are its psychophysiological effects dependent on the stimulation area? Int. J. Psychophysiol. 161, 64–75. doi: 10.1016/j.ijpsycho.2021.01.003, 33444689

[ref14] Brady M. C. Kelly H. Godwin J. Enderby P. Campbell P. (2016). Speech and language therapy for aphasia following stroke. Cochrane Database Syst. Rev. 2016:Cd000425. doi: 10.1002/14651858.CD000425.pub4, 27245310 PMC8078645

[ref15] Burger A. M. D’Agostini M. Verkuil B. Van Diest I. (2020). Moving beyond belief: a narrative review of potential biomarkers for transcutaneous vagus nerve stimulation. Psychophysiology 57:e13571. doi: 10.1111/psyp.13571, 32202671

[ref16] Butt M. F. Albusoda A. Farmer A. D. Aziz Q. (2020). The anatomical basis for transcutaneous auricular vagus nerve stimulation. J. Anat. 236, 588–611. doi: 10.1111/joa.13122, 31742681 PMC7083568

[ref17] Butters E. Collins-Jones L. Mesquita R. C. Acharya D. McKiernan E. Laurell A. A. . (2025). Brain network analysis in Alzheimer’s disease and mild cognitive impairment using high-density diffuse optical tomography. bioRxiv 2025.04. 28.651132. doi: 10.1101/2025.04.28.651132 PMC13112212 42052506

[ref18] Camargo L. Gianlorenço A. C. Pacheco-Barrios K. Pichardo E. Costa V. Choi H. . (2025). The effects of non-invasive transcutaneous auricular vagus nerve stimulation on resting-state delta oscillation: a randomized, double-blinded, sham-control trial. Sci. Rep. 15:34531. doi: 10.1038/s41598-025-17821-5, 41044114 PMC12494723

[ref19] Castro N. Hula W. D. Ashaie S. A. (2023). Defining aphasia: content analysis of six aphasia diagnostic batteries. Cortex 166, 19–32. doi: 10.1016/j.cortex.2023.05.005, 37295235 PMC10560591

[ref20] Chen C. H. Chang T. Y. Sung P. S. Su H.-C. Chou C.-H. Tung H. . (2025). An overview of post-stroke disability. J. Formos. Med. Assoc. 125, S32–S47. doi: 10.1016/j.jfma.2025.10.038, 41203544

[ref21] Cintoli S. Favilli L. Morganti R. Siciliano G. Ceravolo R. Tognoni G. (2024). Verbal fluency patterns associated with the amnestic conversion from mild cognitive impairment to dementia. Sci. Rep. 14:2029. doi: 10.1038/s41598-024-52562-x, 38263387 PMC10806051

[ref22] Dawson J. Liu C. Y. Francisco G. E. Cramer S. C. Wolf S. L. Dixit A. . (2021). Vagus nerve stimulation paired with rehabilitation for upper limb motor function after ischaemic stroke (VNS-REHAB): a randomised, blinded, pivotal, device trial. Lancet 397, 1545–1553. doi: 10.1016/S0140-6736(21)00475-X, 33894832 PMC8862193

[ref23] Dick A. S. Garic D. Graziano P. Tremblay P. (2019). The frontal aslant tract (FAT) and its role in speech, language and executive function. Cortex 111, 148–163. doi: 10.1016/j.cortex.2018.10.015, 30481666 PMC6461388

[ref24] du Boisgueheneuc F. Levy R. Volle E. Boisgueheneuc F. d. Seassau M. Duffau H. . (2006). Functions of the left superior frontal gyrus in humans: a lesion study. Brain 129, 3315–3328. doi: 10.1093/brain/awl244, 16984899

[ref25] Eggebrecht A. T. White B. R. Ferradal S. L. Chen C. Zhan Y. Snyder A. Z. . (2012). A quantitative spatial comparison of high-density diffuse optical tomography and fMRI cortical mapping. NeuroImage 61, 1120–1128. doi: 10.1016/j.neuroimage.2012.01.124, 22330315 PMC3581336

[ref26] Fiske A. de Klerk C. Lui K. Y. K. Collins-Jones L. Hendry A. Greenhalgh I. . (2022). The neural correlates of inhibitory control in 10-month-old infants: a functional near-infrared spectroscopy study. NeuroImage 257:119241. doi: 10.1016/j.neuroimage.2022.119241, 35537598 PMC7616317

[ref27] Forte G. Favieri F. Leemhuis E. De Martino M. L. Giannini A. M. De Gennaro L. . (2022). Ear your heart: transcutaneous auricular vagus nerve stimulation on heart rate variability in healthy young participants. PeerJ. 10:e14447. doi: 10.7717/peerj.14447, 36438582 PMC9686410

[ref28] Gao F. Wang L. Wang Z. Tian Y. Wu J. Wang M. . (2025). Case report: monitoring consciousness with fNIRS in a patient with prolonged reduced consciousness following hemorrhagic stroke undergoing adjunct taVNS therapy. Front. Neurosci. 19:1519939. doi: 10.3389/fnins.2025.1519939, 39967804 PMC11832507

[ref29] Gianlorenco A. C. L. de Melo P. S. Marduy A. Kim A. Y. Kim C. K. Choi H. . (2022). Electroencephalographic patterns in taVNS: a systematic review. Biomedicine 10:2208. doi: 10.3390/biomedicines10092208, 36140309 PMC9496216

[ref30] Gilmore N. Yücel M. A. Li X. Boas D. A. Kiran S. (2021). Investigating language and domain-general processing in Neurotypicals and individuals with aphasia—a functional near-infrared spectroscopy pilot study. Front. Hum. Neurosci. 15:728151. doi: 10.3389/fnhum.2021.728151, 34602997 PMC8484538

[ref31] Hartwig V. Carbonaro N. Tognetti A. Vanello N. (2017). Systematic review of fMRI compatible devices: design and testing criteria. Ann. Biomed. Eng. 45, 1819–1835. doi: 10.1007/s10439-017-1853-1, 28550499

[ref32] Heim S. Eickhoff S. B. Amunts K. (2008). Specialisation in Broca's region for semantic, phonological, and syntactic fluency? NeuroImage 40, 1362–1368. doi: 10.1016/j.neuroimage.2008.01.009, 18296070

[ref33] Herrmann M. J. Horst A. K. Löble S. Möll M. T. Katzorke A. Polak T. (2017). Relevance of dorsolateral and frontotemporal cortex on the phonemic verbal fluency—a fNIRS-study. Neuroscience 367, 169–177. doi: 10.1016/j.neuroscience.2017.10.028, 29111357

[ref34] Holowacz M. Illingworth C. Swalwell J. Blackburn D. Ali A. Majid A. . (2026). Transcutaneous auricular vagus nerve stimulation during a 3-back task: a high-density functional near-infrared spectroscopy study. Exp. Brain Res. 244:156. doi: 10.1007/s00221-026-07354-2, 42437406 PMC13357519

[ref35] Huppert T. J. Diamond S. G. Franceschini M. A. Boas D. A. (2009). HomER: a review of time-series analysis methods for near-infrared spectroscopy of the brain. Appl. Opt. 48, D280–D298. doi: 10.1364/ao.48.00d280, 19340120 PMC2761652

[ref36] Jahani S. Setarehdan S. K. Boas D. A. Yücel M. A. (2018). Motion artifact detection and correction in functional near-infrared spectroscopy: a new hybrid method based on spline interpolation method and Savitzky-Golay filtering. Neurophotonics 5:015003. doi: 10.1117/1.NPh.5.1.015003, 29430471 PMC5803523

[ref37] Kunii N. Koizumi T. Kawai K. Shimada S. Saito N. (2021). Vagus nerve stimulation amplifies task-induced cerebral blood flow increase. Front. Hum. Neurosci. 15:726087. doi: 10.3389/fnhum.2021.726087, 34434098 PMC8380847

[ref38] Li H. Liu J. Tian S. Fan S. Wang T. Qian H. . (2022). Language reorganization patterns in global aphasia-evidence from fNIRS. Front. Neurol. 13:1025384. doi: 10.3389/fneur.2022.1025384, 36686505 PMC9853054

[ref39] Li S. Y. Xu K. Wang Y. X. Wang M. H. Li S. S. Lin F. . (2025). Task-specific cortical mechanisms of taVNS-paired task-oriented training for post-stroke upper extremity dysfunction under cognitive load: an fNIRS study. Front. Hum. Neurosci. 19:1652612. doi: 10.3389/fnhum.2025.1652612, 41070188 PMC12504255

[ref40] Maintz J. B. Viergever M. A. (1998). A survey of medical image registration. Med. Image Anal. 2, 1–36. doi: 10.1016/s1361-8415(01)80026-8, 10638851

[ref41] Matsuoka M. Yamaguchi T. Fujiwara T. (2025). Transcutaneous auricular vagus nerve stimulation in healthy individuals, stroke, and Parkinson's disease: a narrative review of safety, parameters, and efficacy. Front. Physiol. 16:1693907. doi: 10.3389/fphys.2025.1693907, 41211140 PMC12593472

[ref42] Meyers E. C. Solorzano B. R. James J. Ganzer P. D. Lai E. S. Rennaker R. L. II . (2018). Vagus nerve stimulation enhances stable plasticity and generalization of stroke recovery. Stroke 49, 710–717. doi: 10.1161/strokeaha.117.019202, 29371435 PMC6454573

[ref43] Miyatsu T. Oviedo V. Reynaga J. Karuzis V. P. Martinez D. O’Rourke P. . (2024). Transcutaneous cervical vagus nerve stimulation enhances second-language vocabulary acquisition while simultaneously mitigating fatigue and promoting focus. Sci. Rep. 14:17177. doi: 10.1038/s41598-024-68015-4, 39060415 PMC11282064

[ref44] Onishi A. Furutani H. Hiroyasu T. Hiwa S. (2019). An fNIRS study of brain state during letter and category fluency tasks. J. Robot. Netw. Artif. Life 5, 228–231. doi: 10.2991/jrnal.k.190220.003

[ref45] Peuker E. T. Filler T. J. (2002). The nerve supply of the human auricle. Clin. Anat. 15, 35–37. doi: 10.1002/ca.1089, 11835542

[ref46] Phillips I. Calloway R. C. Karuzis V. P. Pandža N. B. O'Rourke P. Kuchinsky S. E. (2021). Transcutaneous auricular Vagus nerve stimulation strengthens semantic representations of foreign language tone words during initial stages of learning. J. Cogn. Neurosci. 34, 127–152. doi: 10.1162/jocn_a_01783, 34673939

[ref47] Pinti P. Tachtsidis I. Hamilton A. Hirsch J. Aichelburg C. Gilbert S. . (2020). The present and future use of functional near-infrared spectroscopy (fNIRS) for cognitive neuroscience. Ann. N. Y. Acad. Sci. 1464, 5–29. doi: 10.1111/nyas.13948, 30085354 PMC6367070

[ref48] Price C. J. (2010). The anatomy of language: a review of 100 fMRI studies published in 2009. Ann. N. Y. Acad. Sci. 1191, 62–88. doi: 10.1111/j.1749-6632.2010.05444.x, 20392276

[ref49] Rolls E. T. (2023). Emotion, motivation, decision-making, the orbitofrontal cortex, anterior cingulate cortex, and the amygdala. Brain Struct. Funct. 228, 1201–1257. doi: 10.1007/s00429-023-02644-9, 37178232 PMC10250292

[ref50] Rolls E. T. Joliot M. Tzourio-Mazoyer N. (2015). Implementation of a new parcellation of the orbitofrontal cortex in the automated anatomical labeling atlas. NeuroImage 122, 1–5. doi: 10.1016/j.neuroimage.2015.07.075, 26241684

[ref51] Scarapicchia V. Brown C. Mayo C. Gawryluk J. R. (2017). Functional magnetic resonance imaging and functional near-infrared spectroscopy: insights from combined recording studies. Front. Hum. Neurosci. 11:419. doi: 10.3389/fnhum.2017.00419, 28867998 PMC5563305

[ref52] Srinivasan S. Acharya D. Butters E. Collins-Jones L. Mancini F. Bale G. (2024). Subject-specific information enhances spatial accuracy of high-density diffuse optical tomography. Front Neuroergon. 5:1283290. doi: 10.3389/fnrgo.2024.1283290, 38444841 PMC10910052

[ref53] Sun J. Li X. Zhou Y. Liu H. Ma C. Hao C. . (2025). Clinical advances in transcutaneous auricular vagus nerve stimulation for post-stroke disorders: state of the art and future perspectives. Front. Neurol. 16:1676727. doi: 10.3389/fneur.2025.1676727, 41245869 PMC12615202

[ref54] Tachtsidis I. Scholkmann F. (2016). False positives and false negatives in functional near-infrared spectroscopy: issues, challenges, and the way forward. Neurophotonics 3:031405. doi: 10.1117/1.NPh.3.3.031405, 27054143 PMC4791590

[ref55] Tupak S. V. Badewien M. Dresler T. Hahn T. Ernst L. H. Herrmann M. J. . (2012). Differential prefrontal and frontotemporal oxygenation patterns during phonemic and semantic verbal fluency. Neuropsychologia 50, 1565–1569. doi: 10.1016/j.neuropsychologia.2012.03.009, 22426205

[ref56] Uchitel J. Blanco B. Vidal-Rosas E. Collins-Jones L. Cooper R. J. (2022). Reliability and similarity of resting state functional connectivity networks imaged using wearable, high-density diffuse optical tomography in the home setting. NeuroImage 263:119663. doi: 10.1016/j.neuroimage.2022.119663, 36202159

[ref57] von Lühmann A. Li X. Müller K. R. Boas D. A. Yücel M. A. (2020). Improved physiological noise regression in fNIRS: a multimodal extension of the general linear model using temporally embedded canonical correlation analysis. NeuroImage 208:116472. doi: 10.1016/j.neuroimage.2019.116472, 31870944 PMC7703677

[ref58] Wagner S. Sebastian A. Lieb K. Tüscher O. Tadić A. (2014). A coordinate-based ALE functional MRI meta-analysis of brain activation during verbal fluency tasks in healthy control subjects. BMC Neurosci. 15:19. doi: 10.1186/1471-2202-15-19, 24456150 PMC3903437

[ref59] Wang Z. Cheng K. Bai J. Zhang C. Ni J. (2025). Feasibility of vagus nerve modulation (VNMM/taVNS) in cognitive-swallowing dual-task paradigms: a fNIRS pilot study. Front. Neurosci. 19:1619532. doi: 10.3389/fnins.2025.1619532, 40860847 PMC12375658

[ref60] Wang L. Gao F. Dai Y. Wang Z. Liang F. Wu J. . (2023). Transcutaneous auricular vagus nerve stimulation on upper limb motor function with stroke: a functional near-infrared spectroscopy pilot study. Front. Neurosci. 17:1297887. doi: 10.3389/fnins.2023.1297887, 38075278 PMC10702495

[ref61] Wang M. H. Jin Y. J. He M. F. Zhou A. N. Zhu M. L. Lin F. . (2024). Transcutaneous auricular vagus nerve stimulation improves cognitive decline by alleviating intradialytic cerebral hypoxia in hemodialysis patients: a fNIRS pilot study. Heliyon 10:e39841. doi: 10.1016/j.heliyon.2024.e39841, 39975458 PMC11838084

[ref62] Wroblewski G. J. Matsuo K. Hirata K. Matsubara T. Harada K. Watanabe Y. . (2017). Effects of task language and second-language proficiency on the neural correlates of phonemic fluency in native Japanese speakers: a functional near-infrared spectroscopy study. Neuroreport 28, 884–889. doi: 10.1097/WNR.0000000000000852, 28763376

[ref63] Yakunina N. Kim S. S. Nam E. C. (2017). Optimization of transcutaneous Vagus nerve stimulation using functional MRI. Neuromodulation 20, 290–300. doi: 10.1111/ner.12541, 27898202

[ref64] Zhu S. Liu Q. Zhang X. Zhou M. Zhou X. Ding F. . (2024). Transcutaneous auricular vagus nerve stimulation enhanced emotional inhibitory control via increasing intrinsic prefrontal couplings. Int. J. Clin. Health Psychol. 24:100462. doi: 10.1016/j.ijchp.2024.100462, 38665809 PMC11044052

