# Supplementary material for: Transcutaneous vagus nerve stimulation during semantic verbal fluency tasks: a high-density diffuse optical tomography study
Source: Front Hum Neurosci. 2026 Oct 5;20:1964706. doi: 10.3389/fnhum.2026.1964706 (PMC13677701; doi:10.3389/fnhum.2026.1964706)
Supplement: Supplementary file 1 [file Supplementary_file_1.docx]

**Appendix A**

**Figure A1.**  Automated Anatomical Labelling (AAL2) atlas Cortical regions for group-level parcellation analysis.

**Appendix B- Performance effects of taVNS**

To investigate the effect of stimulation on the relationship between activation and performance, we ran a Pearson correlational analysis between the ΔHbO concentration seen in each parcel, and participants’ mean VFT score for each stimulation condition. Given the lack of a priori hypothesis, an FDR correction was applied to reduce the risk of Type I error. As with the behavioural analysis, two participants were not included in the analysis due to missing data. A heatmap of these correlations are shown in Figure B1.

No significant association was revealed between task-evoked ΔHbO within parcels and the mean number of words produced across VFT trials in the Sham taVNS condition, nor when looking at the differences in performance (active-minus-sham score) relative to activation (active-minus-sham ΔHbO) across stimulation conditions. In contrast, during active stimulation, there was a significant positive correlation between HbO concentration increase and the mean VFT score in the left Anterior orbital gyrus (OFCant_L) (r = 0.53, 95% CI [0.13, 0.78], n = 21, p = .014) and the left Dorsolateral superior frontal gyrus (Frontal_Sup_2_L) (r = 0.48, 95% CI [0.08, 0.75], n = 22, p = .021). However, neither of these correlations survived FDR correction (OFCant_L FDR = .11; Frontal_Sup_2_L FDR = .13).

As such, taVNS may support the more effective use of different brain regions, wherein the activation is not significantly different from sham (as seen in the parcellation table) but more effectively supports task performance. Nonetheless, given the absence of association between the difference in relative ΔHbO and performance between the two stimulation conditions, we can see no significant effect of TaVNS on performance nor the underlying activity supporting this after correcting for multiple correlations.

**Figure B1**. Correlation heatmaps between the change in HbO concentration from baseline to task in each parcel and mean number of correct responses during verbal fluency tasks. * denotes uncorrected *p*<.05.

**Appendix C- Full Parcellation results**

|  | **Parcels** | **N** | **Baseline HbO (µM)** | | **Task HbO (µM)** | | **Δ HbO (µM)** | ***p*** | **FDR *p*** | **Cohen’s *d*** |
| --- | --- | --- | --- | --- | --- | --- | --- | --- | --- | --- |
|  |  |  |  |  |  |  |  |  |  |  |
|  |  |  | ***M*** | ***SD*** | ***M*** | ***SD*** |  |  |  |  |
| VFT Sham | Frontal_Sup_2_L | 23 | -4.70 E-18 | 1.31 E-17 | 0.07 | 0.13 | 0.07 | .**02** | .08 | 0.52 |
|  | Frontal_Sup_2_R | 21 | 2.13 E-18 | 1.03 E-17 | 0.07 | 0.17 | 0.07 | .08 | .20 | 0.41 |
|  | Frontal_Mid_2_L | 22 | 9.73 E-20 | 7.72 E-18 | 0.05 | 0.09 | 0.05 | .**02** | .08 | 0.53 |
|  | Frontal_Mid_2_R | 22 | 3.36 E-18 | 7.87 E-18 | 0.05 | 0.14 | 0.05 | .13 | .27 | 0.34 |
|  | Frontal_Inf_Tri_L | 17 | 1.05 E-18 | 1.07 E-17 | 0.10 | 0.13 | 0.10 | .**01** | .05 | 0.75 |
|  | Frontal_Inf_Tri_R | 20 | 1.53 E-18 | 7.64 E-18 | 0.02 | 0.09 | 0.02 | .35 | .48 | 0.21 |
|  | Frontal_Inf_Orb_2_R | 22 | -3.85 E-18 | 7.20 E-18 | 0.01 | 0.13 | 0.01 | .78 | .83 | 0.06 |
|  | Frontal_Sup_Medial_L | 22 | -2.32 E-19 | 5.55 E-18 | 0.02 | 0.12 | 0.02 | .51 | .59 | 0.14 |
|  | Frontal_Sup_Medial_R | 23 | 5.77 E-19 | 6.63 E-18 | 0.05 | 0.13 | 0.05 | .08 | .20 | 0.39 |
|  | Frontal_Med_Orb_L | 24 | 3.57 E-18 | 1.38 E-17 | 0.03 | 0.18 | 0.03 | .40 | .49 | 0.18 |
|  | Frontal_Med_Orb_R | 18 | -1.68 E-18 | 7.73 E-18 | 0.00 | 0.10 | 0.00 | .89 | .89 | -0.03 |
|  | OFCant_L | 21 | -5.67 E-19 | 5.25 E-18 | 0.02 | 0.09 | 0.02 | .25 | .38 | 0.26 |
|  | OFCant_R | 20 | -2.48 E-18 | 8.83 E-18 | -0.02 | 0.07 | -0.02 | .17 | .32 | -0.32 |
|  | OFClat_L | 24 | 4.74 E-19 | 8.45 E-18 | 0.10 | 0.14 | 0.10 | .**002** | .**03*** | 0.71 |
|  | OFClat_R | 18 | -8.55 E-19 | 4.40 E-18 | -0.02 | 0.05 | -0.02 | .22 | .36 | -0.30 |
| VFT Active | Frontal_Sup_2_L | 23 | 1.17 E-18 | 7.40 E-18 | 0.03 | 0.12 | 0.03 | .23 | .67 | 0.26 |
|  | Frontal_Sup_2_R | 21 | 5.66 E-19 | 6.53 E-18 | 0.00 | 0.14 | 0.00 | .91 | .97 | 0.02 |
|  | Frontal_Mid_2_L | 22 | 1.64 E-18 | 5.31 E-18 | 0.02 | 0.13 | 0.02 | .45 | .75 | 0.16 |
|  | Frontal_Mid_2_R | 22 | -1.54 E-18 | 8.76 E-18 | 0.01 | 0.12 | 0.01 | .78 | .97 | 0.06 |
|  | Frontal_Inf_Tri_L | 17 | 3.08 E-19 | 4.48 E-18 | 0.06 | 0.09 | 0.06 | .**02** | .23 | 0.66 |
|  | Frontal_Inf_Tri_R | 20 | -1.36 E-18 | 4.99 E-18 | -0.01 | 0.07 | -0.01 | .33 | .67 | -0.22 |
|  | Frontal_Inf_Orb_2_R | 22 | 2.50 E-21 | 5.55 E-18 | -0.02 | 0.13 | -0.02 | .57 | .85 | -0.12 |
|  | Frontal_Sup_Medial_L | 22 | -2.59 E-19 | 4.16 E-18 | 0.03 | 0.11 | 0.03 | .15 | .67 | 0.32 |
|  | Frontal_Sup_Medial_R | 23 | -3.17 E-19 | 6.13 E-18 | 0.00 | 0.13 | 0.00 | .97 | .97 | 0.01 |
|  | Frontal_Med_Orb_L | 24 | 6.38 E-18 | 1.25 E-17 | 0.00 | 0.13 | 0.00 | .87 | .97 | -0.03 |
|  | Frontal_Med_Orb_R | 18 | 2.28 E-19 | 3.98 E-18 | -0.03 | 0.08 | -0.03 | .11 | .67 | -0.39 |
|  | OFCant_L | 21 | 3.70 E-19 | 3.89 E-18 | -0.02 | 0.11 | -0.02 | .36 | .67 | -0.21 |
|  | OFCant_R | 20 | -1.29 E-18 | 6.87 E-18 | -0.03 | 0.10 | -0.03 | .19 | .67 | -0.30 |
|  | OFClat_L | 24 | 1.67 E-18 | 1.14 E-17 | 0.01 | 0.15 | 0.01 | .72 | .97 | 0.07 |
|  | OFClat_R | 18 | 5.05 E-19 | 2.78 E-18 | -0.01 | 0.04 | -0.01 | .27 | .67 | -0.27 |

**Table C1.** Parcel-Wise Statistical Comparison of HbO Concentration Changes during Verbal Fluency Tasks (All Parcels).

Key - Frontal_Sup_2 (left/right) – superior frontal gyrus (dorsolateral); Frontal_Mid_2 (left/right) – middle frontal gyrus (dorsolateral); Frontal_Inf_Tri (left/right) – inferior frontal gyrus, trian-gular part; Frontal_Inf_Orb_2_R – inferior frontal gyrus, orbital part (right); Frontal_Sup_Medial (left/right) – superior medial frontal gyrus; Frontal_Med_Orb (left/right) – medial orbital frontal cortex; OFCant (left/right) – anterior orbitofrontal cortex; and OFClat (left/right) – lateral or-bitofrontal cortex.

**Appendix D- Handedness Subanalysis**

Due to the heterogeneity of the handedness and language background of the cohort included, we also ran equivalent t-tests looking at the HbO concentration change from baseline (-5 to 0 seconds) to task (10-20 seconds) across stimulation conditions in sub cohorts defined as: only monolinguals, only right handers, and only right-handed monolinguals. Uncorrected t-statistic maps are shown in Figure D1. The patterns of activation of these cohort were consistent with the patterns seen across the whole group when all cohorts were included, with a deactivation in the left posterior frontal regions during active compared to sham condition (Figure 5).

**Figure D1.** Cortical surface reconstruction of t-statistic maps comparing the block-averaged HbO concentration at baseline (-5 to 0 seconds pre-task) vs task-peak (10 to 20 seconds) at the node-level (uncorrected for multiple comparisons) in each subgroup. Only nodes with uncorrected p<.05 are shown.
